# Machine Learning Techniques to Analyze the Influence of Silica on the Physico-Chemical Properties of Aerogels

**DOI:** 10.3390/gels10090554

**Published:** 2024-08-27

**Authors:** Hamdi Chaouk, Emil Obeid, Jalal Halwani, Jack Arayro, Rabih Mezher, Omar Mouhtady, Eddie Gazo-Hanna, Semaan Amine, Khaled Younes

**Affiliations:** 1 College of Engineering and Technology, American University of the Middle East, Egaila 54200, Kuwait; hamdi-chaouk@aum.edu.kw (H.C.); jack.arayro@aum.edu.kw (J.A.); rabih.mezher@aum.edu.kw (R.M.); omar.mouhtady@aum.edu.kw (O.M.); eddie-hanna@aum.edu.kw (E.G.-H.); semaan.amine@aum.edu.kw (S.A.); 2 Water and Environment Sciences Laboratory, Lebanese University, Tripoli P.O. Box 6573/14, Lebanon; jhalwani@ul.edu.lb

**Keywords:** silica aerogels, composite, principal component analysis, physical properties

## Abstract

This study explores the application of machine learning techniques, specifically principal component analysis (PCA), to analyze the influence of silica content on the physical and chemical properties of aerogels. Silica aerogels are renowned for their exceptional properties, including high porosity, large surface area, and low thermal conductivity, but their mechanical brittleness poses significant challenges. The study initially utilized cross-correlation analysis to examine the relationships between key properties such as the Brunauer–Emmett–Teller (BET) surface area, pore volume, density, and thermal conductivity. However, weak correlations prompted the application of PCA to uncover deeper insights into the data. The PCA results demonstrated that silica content has a significant impact on aerogel properties, with the first principal component (PC1) showing a strong positive correlation (R^2^ = 94%) with silica content. This suggests that higher silica levels correspond to lower thermal conductivity, porosity, and BET surface area, while increasing the density and elastic modulus. Additionally, the analysis identified the critical role of thermal conductivity in the second principal component (PC2), particularly in samples with moderate to high silica content. Overall, this study highlights the effectiveness of machine learning techniques like PCA in optimizing and understanding the complex inter-relationships among the physico-chemical properties of silica aerogels.

## 1. Introduction

Inorganic silica aerogel (SA) is an amorphous material renowned for its remarkable properties, which include high porosity, a large surface area, ultra-low density, and low thermal conductivity [1]. These attributes predominantly arise from its intricate three-dimensional porous silica matrix network [2], creating an extensive network of interconnected nanostructures. Due to these unique characteristics, silica aerogels find applications across a wide range of fields [3,4]. For instance, their exceptional thermal insulation capabilities make them ideal for use in industries requiring advanced thermal management [5,6,7]. Silica aerogels are commonly used in building materials to improve energy efficiency by reducing heat transfer [8], which is particularly valuable in both residential and commercial construction [2]. Additionally, their lightweight nature, combined with excellent insulating properties, makes them suitable for aerospace applications [9], where reducing weight without compromising insulation is critical. Furthermore, their extensive surface area and high porosity are advantageous in catalyst support applications [10]. In chemical processes, silica aerogels provide a large surface area for catalysts to disperse, enhancing reaction efficiency and improving overall process performance. This makes them a preferred choice in petrochemical industries and other sectors where catalytic reactions are pivotal [11]. Environmental depollution processes also benefit significantly from the properties of silica aerogels [12]. Due to their high surface area and porosity, silica aerogels are highly effective in absorbing and adsorbing pollutants from air and water [13]. This includes the removal of heavy metals, organic contaminants, and other hazardous substances, making them a valuable tool in environmental cleanup and pollution control efforts [14].

However, the open porous structure of the silica matrix exhibits low mechanical strength and brittleness, which limits its use in many applications [15]. Therefore, enhancing the mechanical strength while optimizing the physico-chemical properties of SA is crucial [16]. SA has been developed with fillers such as fibers, polymers, and metal oxides to expand their applications. The silica aerogel matrix facilitates easy reinforcement strategies through cross-linking with different fillers [17,18], significantly benefiting the reinforced silica hydrogel composites by enhancing their mechanical properties while retaining the desirable features of native silica aerogels. The incorporation of nanoparticles [18], fibers [19], or polymers as binders to improve thermal, mechanical, or optical properties has been extensively studied over the past decade [14]. Several researchers have focused on the synergistic effects on the physico-chemical properties of silica aerogel composites. Guzel et al. [20] reviewed the synergistic effects on the properties of polymer composites of silica aerogels/xerogels. Karamikamkar et al. evaluated the role of precursors in producing silica-based aerogels to enhance mechanical strength, modify morphology, and increase multifunctionality [21]. The incorporation of fillers such as nanoparticles [18], fibers [22], polymers, and metal oxides pose significant challenges in optimizing the physico-chemical properties of silica aerogel for widespread application. While these fillers hold promise in enhancing the specific characteristics of silica aerogels, their integration complicates the overall optimization process [23]. Each filler introduces its own set of complexities, influencing the structural integrity, porosity, thermal conductivity, and mechanical strength of the aerogel matrix [23]. Moreover, achieving a balanced combination of these properties is an intricate process, as the interactions between the silica matrix and fillers must be carefully managed to avoid unintended consequences. Despite advancements in material science and engineering [9], the precise control and fine-tuning required to harness the full potential of silica aerogel composites remain formidable tasks. Consequently, while the incorporation of fillers offers avenues for tailoring silica aerogels to meet diverse application requirements [9], the intricate optimization process presents barriers to their widespread utilization across various industries and domains.

Thus, the study of the interconnections between the various physico-chemical properties of SA can reveal its suitability for numerous important applications [15]. For this purpose, principal component analysis (PCA) is widely used in various applications, including electrocatalysis for green hydrogen production [24], water treatment [25], and desalination [25]. In the context of electrocatalysis for green hydrogen production, PCA has been employed to investigate the features of aerogel-based electrocatalysts. Critical parameters influencing the catalytic activity in water-splitting reactions were identified by analyzing the correlation between the characteristics of these electrocatalysts and their related overpotential [24]. Conversely, acquiring a reliable water supply for urban and industrial use is one of the greatest challenges for sustainability. PCA has proven to be a valuable tool in the field of water desalination, where it has been successfully applied in multiple studies to optimize desalination processes. It helped to identify the critical factors and interrelationships that improve the efficiency and effectiveness of desalination technologies [25]. This application of PCA highlights its capability to manage complex datasets and provide valuable insights for enhancing membrane technologies in water desalination [25]. Nonetheless, several challenges appear following the application of PCA to a set of materials. The most significant one is the low variance that could occur from the application of PCA to the row data. In order to overcome this problem, data normalization and reduction techniques could be utilized [25,26,27,28,29].

In this study, a principal machine learning model was employed to study the cross-correlation among the examined physico-chemical properties of silica aerogel, including ‘wt.% vs. silica’, ‘BET’, ‘pore volume’, ‘average pore diameter’, ‘density’, ‘thermal conductivity’, ‘porosity’, and ‘elastic modulus’. The observed correlations were weak and did not provide a meaningful covariance between these features, as shown in this work. Therefore, principal component analysis (PCA) was used to reduce the number of physico-chemical properties of the silica aerogel, thereby uncovering the complex interrelations among these properties. Our aim was to understand how these properties interact and influence the aerogel’s suitability for diverse applications [3]. Following PCA analysis, the yielded output would be compared with the amount of nanosilica in aerogels, in order to confirm or refute the influence of the latter on the physical and mechanical features. This comparison would be made by regression analysis. While PCA proved instrumental in providing insights into these interrelations [24,30], our findings also shed light on the challenges posed by the intricate optimization process. Despite the potential of silica aerogels to meet a wide range of application requirements, the complexity of optimizing their properties presents significant barriers to their widespread utilization across various industries and domains.

## 2. Results and Discussion

### 2.1. Principal Machine Learning Model for Exploring Cross-Correlations among the Physico-Chemical Properties of Silica Aerogel

**Figure 1:**
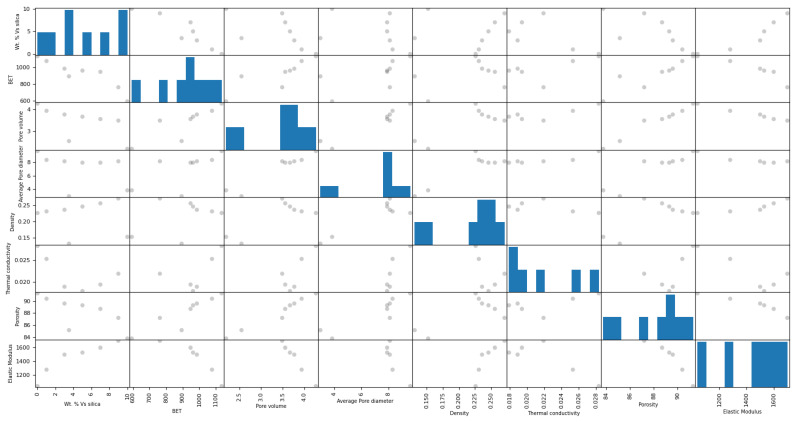
Cross-correlations among the physico-chemical properties of silica aerogel.

To assess the cross-correlation among various chemical and mechanical properties of silica aerogel, including features such as ‘wt.% vs. silica’, ‘BET’, ‘pore volume’, ‘average pore diameter’, ‘density’, ‘thermal conductivity’, ‘porosity’, and ‘elastic modulus’, we utilized a cross-correlation matrix analysis machine learning model (Figure 1). For instance, the bottom left panel of Figure 1 illustrates a positive correlation between the elastic modulus and the wt.% of silica. However, the observed correlations were weak and did not yield meaningful covariance among the investigated properties for the silica aerogel being studied. To obtain more insightful correlations, and leverage qualitative judgments, it is necessary to reduce the dimensionality of the investigated dataset. Hence, principal component analysis (PCA) is found to be an adequate tool for implementation. This will enable simplifying the set of properties and reveal the complex interrelations among them.

### 2.2. Principal Component Analysis for Silica Aerogel Composite

**Figure 2:**
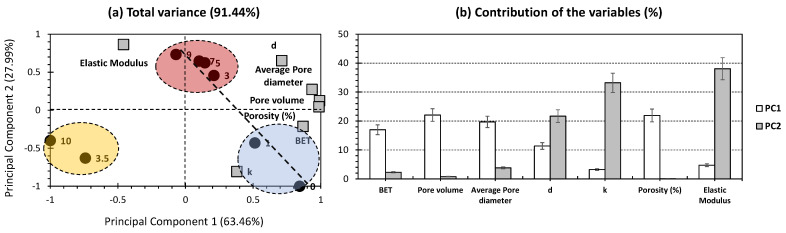
(**a**) PCA biplot representation of datasets for the properties of silica aerogel composites that possess TEOS as a precursor and glass fiber as a reinforcing agent (data were obtained from the previous findings of Jadhav and Sarawade [14]; Table 1). Black circular bullets indicate silica aerogels following the wt.% of silica; square grey bullets indicate the physical properties involved. (**b**) Contribution of the variables (%) following the first two PCs.

Figure 2 shows the PCA biplot carried out with the dataset of the properties of silica aerogel composite drawn from the previously collected findings of Jadhav and Sarawade [14]. Remarkably, a high presentation of total variance has been recorded, scoring 91.44% (63.46% and 27.99% for PC1 and PC2, respectively; Figure 2a). This indicates that PCA was highly effective in presenting most of the trends between the investigated aerogels with only two dimensions. This reflects the high efficiency and reliability of the statistical method in showing correlations and discrepancies between aerogels and the relative physical properties being studied. In previous attempts, most of the total variance yielded around 60–88%, even with several pre-treatments, with the removal of outliers [26,28,29,31]. In the scope of the physical properties being studied (variables of the PCA; Figure 2b), it is intriguing to highlight that most of them exhibited independent contributions with respect to the first two PCs. In other words, variables that made a contribution to PC1 made a negligible contribution to PC2, and vice versa. The only exception was for density (d), as it scored moderate contributions to both PCs (11.37% and 21.7% for PC1 and PC2, respectively; Figure 2b). In the case of PC1, all variables, with the exception of thermal conductivity (k) and elastic modulus, made moderate contributions (10–25%). Conversely, the major contributions for thermal conductivity (k) and elastic modulus were made to PC2 (33.21% and 38.08%, respectively; Figure 2b).

For the individual samples (aerogel composites with different wt.% of silica), three different clusters can be distinguished (Figure 2a). Interestingly, the yellow cluster exclusively encompassed samples of aerogels dried under atmospheric pressure conditions (samples holding 3.5% and 10% of wt.% silica; Table 1 and Figure 2a). These samples exhibited negative trends for both PCs and were plotted away from the different variables being studied. For the aerogel samples dried under supercritical conditions, they were located between the blue and red clusters. The first samples garnered a negligible and low wt.% of silica, and the second garnered average to high loads. The blue cluster samples were plotted on the positive and negative sides of PC1 and PC2, respectively (Figure 2a). They showed a high influence by BET and thermal conductivity (k). The red cluster samples were plotted at the positive sides of both PCs and showed a high positive influence with respect to the rest of the variables. Interestingly, a high correlation was highlighted between the samples of the blue and the red cluster samples, as they showed a good alignment (Figure 2a). Following the yielded trends, especially with the exclusion of samples of atmospheric pressure drying conditions, a PCA for samples dried under supercritical conditions would contribute to higher trends of correlation between the investigated variables, on one side, and aerogel samples, on the other side.

**Figure 3:**
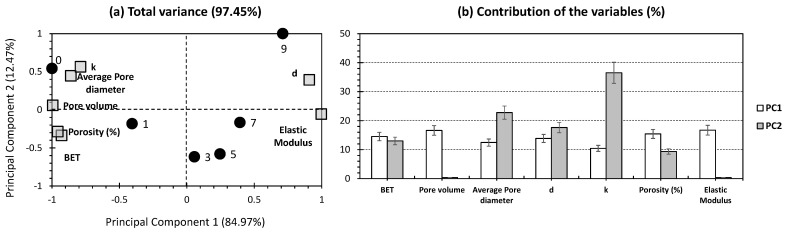
(**a**) PCA biplot representation of the dataset of Table 1, with the exclusion of aerogels dried by APD (3.5% and 10% of the wt.% of silica). Black circular bullets indicate silica aerogels following the wt.% of silica; square grey bullets indicate the physical properties involved. (**b**) Contribution of the variables (%) following the first two PCs.

Figure 3 shows the PCA biplot carried out to exclusively investigate aerogels that were dried under supercritical conditions (excluding aerogels dried by APD (3.5% and 10% of the wt.% of silica). Remarkably, a higher presentation of the total variance has been recorded, scoring 97.45% (84.97% and 12.47% for PC1 and PC2, respectively; Figure 3a). This indicates the efficiency of removing samples dried by APD. The high representativeness of the data set in question would compile more reliable models for the estimation of the effect of nanosilica particles in aerogels. In the scope of the physical properties being studied (variables of the PCA; Figure 3b), it is intriguing to highlight that most of them exhibited moderate contributions to PC1 (around 10–20%). The equally proportional contribution to PC1 indicates the relevance of all investigated variables in showcasing the disparities between the investigated aerogels. For PC2, thermal conductivity scored the highest contribution (36.52%; Figure 3b). Special emphasis has been assigned to thermal conductivity. It can be inferred that thermal conductivity (k) is influenced by physical properties such as the specific surface area (BET), porosity, pore diameter, and volume density (d). Thus, these parameters play key roles in thermal conductivity. Furthermore, Haq et al. [8] minimized the thermal conductivity of their obtained silica aerogels by maintaining a low density, low surface area, and high porosity. To better understand the correlation between k, BET, porosity, pore diameter, and d, we refer to the mathematical definition of the thermal conductivity for silica aerogels. Hence, in [32] and [14], k is, in principle, linked to several parameters: the first is solid thermal conductivity (λs), primarily identified by the material’s density; λs decreases when d is reduced. The second parameter is the gas thermal conductivity (λg), which is primarily governed by the pore diameter; generally, smaller pore diameters result in lower λg. An analysis of Table 1 confirms this, showing that conductivity reduced from 0.0283 W/mK (for an average pore diameter of 9.6 nm) to 0.0179 W/mK (for an average pore diameter of 7.9 nm). Another parameter is fiber thermal conductivity (λf). Elsewhere, Zhang et al. [32] embedded nanofibers in silica aerogel composites, enhancing the thermal insulation. Moreover, the dependency of k on porosity was shown by adopting a dual-mesoporous aerogel structure [32]. This particular structure possessed two advantages: small pores (in the interval of 3 to 6 nm) that diminished the collision frequency of air molecules, thus reducing λg; and large pores (in the range of 17 to 30 nm) that required less supporting framework and, therefore, reduced λs. In conclusion, the overall thermal conductivity was reduced and optimized; not to mention that reducing the thermal conductivity of silica aerogels is highly advantageous since these materials are frequently used in many thermal insulation applications [33].

For PC2, density (d), average pore diameter, and BET showed moderate contributions (17.62%, 22.77%, and 13%, respectively; Figure 3b). The rest of the variables made low to minor contributions to PC2. Following these trends, PC2 would likely emphasize thermal conductivity and its dependent factors (density, average pore diameter, and BET, in our case). Interestingly, no variable has been plotted around the “node”, highlighting the relevance of employing the different physical properties and the reliability of the adopted statistical approach. Conversely, a high correlation between thermal conductivity, porosity, and BET has been found (Figure 3a). The latter group was located on the other side of the PCA biplot with respect to the density and elastic modulus feature (Figure 3a).

For the individual samples (aerogel composites with different wt.% of silica), no significant clustering was apparent (Figure 3a). The only agglomeration noticed was for the 3%, 5%, and 7% samples, which yielded results along the positive and negative sides of PC_1_ and PC_2_, respectively. The exclusion of marginal cases, represented here by 0% and 9% (the minimum and maximum wt.% of silica, respectively), would indicate the high influence of silica on the physical properties of the aerogel composite. The agglomeration for intermediate wt.% cases suggests that no silica or small amounts (represented here by 0% and 1%) were plotted along with most of the variables, indicating a higher degree of magnitude in most of the investigated physical properties. These findings are clear in Table 1, as 0% and 1% samples scored the highest BET specific surface area (1135.4 m^2^/g and 1077.4 m^2^/g, respectively), pore volume (4.27 cm^3^/g and 3.94 cm^3^/g), average pore diameter (9.6 nm and 8.3 nm), and porosity (91.3% and 90.4%). Conversely, the strong deviation noted for density and the elastic modulus, showing a positive influence along the positive side of PC1, and moderate influence along the positive side of PC2, can be confirmed by the lowest values yielded for these two parameters (Table 1). Interestingly, the 3%, 5%, and 7% samples have been plotted between the mentioned marginal cases, showing an intermediate value for the different properties being studied. The highest silica content (9%) was plotted on the positive side of both PCs, with its highest proximity to d and the elastic modulus.

The high correlation between thermal conductivity, porosity, and BET surface area in silica aerogels can be explained through their interconnected physical properties [34,35,36,37]. Aerogels are known for their low thermal conductivity, making them excellent thermal insulators [34,35,36,37]. This low thermal conductivity is primarily due to their high porosity, which reduces the pathways for heat transfer [36]. The air trapped within the porous structure is a poor conductor of heat, contributing significantly to the material’s insulating properties [34]. Porosity represents the measure of the void spaces within the material. High porosity means that a larger volume of the material is made up of air-filled pores rather than solid material [36]. This high porosity is directly linked to low thermal conductivity because it reduces the density and the solid pathways through which heat can travel [36]. This is reflected in our case by the opposite trends between density, from one side, and porosity and thermal conductivity, from the other side (Figure 3a). BET is a measure of the total surface area of a porous material. In the case of aerogels, high porosity typically results in a large BET surface area due to the extensive network of pores and internal surfaces [35]. A larger surface area provides more sites for interactions, which is beneficial in applications such as adsorption and catalysis [38]. While thermal conductivity is primarily influenced by porosity, it indirectly correlates with BET surface area. A higher BET surface area indicates a more complex pore structure, contributing to the reduction of thermal conductivity by disrupting heat flow pathways [34,35,36,37]. This correlation has been translated in our case following the negative influence of both BET and thermal conductivity along PC1 (Figure 3). In brief, the high correlation between thermal conductivity, porosity, and BET surface area in silica aerogels highlights the interdependence of these properties. High porosity contributes to low thermal conductivity and a high BET surface area, making aerogels efficient insulators with significant surface interactions for use in various industrial applications. Improving the mechanical strength of these materials while maintaining these advantageous properties is a critical area of research to expand their applicability.

### 2.3. Principal Components vs. the Percentage of Silica in Aerogel Composites

**Figure 4:**
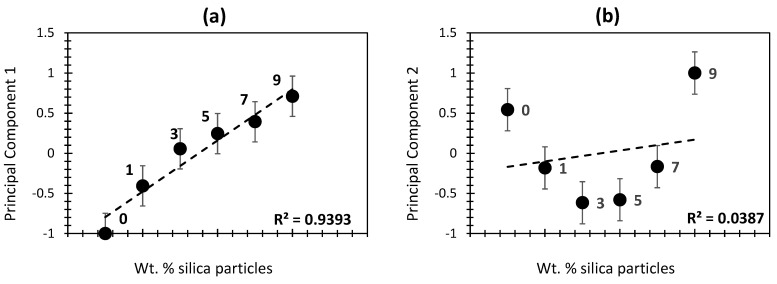
Principal components PC1 (**a**) and PC2 (**b**), with respect to the wt.% of silica in the investigated aerogel composite.

**Figure 5:**
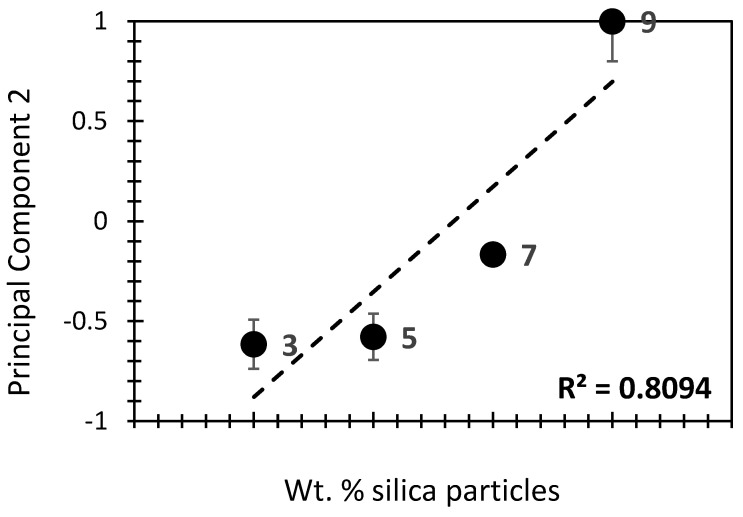
PC2 with respect to the wt.% of silica in the investigated aerogel composite, with the exclusion of samples with a low amount of silica (0% and 1%).

Figure 4 shows the effect of wt.% of silica on the different physical features; these features are represented by PC_1_ (for all variables) and PC_2_ (for thermal conductivity, average pore diameter, and BET surface area, specifically) (Figure 4a,b, respectively). Interestingly, PC_1_ showed a high positive correlation with wt.% of silica (R^2^ = 94 %). This would indicate that the higher the silica content, the lower the thermal conductivity, porosity, and BET surface area will be. Conversely, the density and elastic modulus will be higher along with the silica content. The high correlation yielded in this case would indicate the mutual and equally shared influence of the physical properties being studied. When PC2 is taken into consideration, the low correlation score (R^2^ = 3.87%; Figure 4b) suggests a low influence of silica aerogels on thermal conductivity, in the first place, and on density and average pore diameter, in the second place. In other words, the three latter physical properties cannot fully describe the patterns in silica aerogel composites, highlighting the relevance of adding more physical features, as in the case of the PC_1_ findings. Interestingly, when the low- or no-silica samples (0% and 1%) were excluded, the correlation of PC2 along with the silica amount yielded more coherent results (R^2^ = 81%; Figure 5). This would indicate that thermal conductivity, average pore diameter, and BET surface area are more likely to be influenced by a moderate to high wt.% of silica than those homologs with low or no silica addition. In fact, the influence of silica on the density of aerogel composites is a balance between maintaining a highly porous structure while ensuring sufficient mechanical integrity [4,39,40]. Adjustments in synthesis conditions such as particle size, porosity, and the addition of reinforcing agents allow for optimizing aerogel density to meet specific requirements for various applications [41,42]. In terms of BET surface, silica addition contributes a significant influence through synthesis conditions, surface modifications, and aerogel application [34,43]. By carefully controlling these factors during the synthesis and post-synthesis processes, it is possible to optimize the BET surface area of silica aerogels to meet the requirements of various applications [34,43,44].

## 3. Conclusions

The findings from the principal component analysis (PCA) applied to the silica aerogel composites provide significant insights into the relationships and interdependencies among the physical and chemical properties of these materials. Initially, the study aimed to explore the cross-correlation among various physico-chemical properties, such as the weight percentage of silica, BET surface area, pore volume, average pore diameter, density, thermal conductivity, porosity, and elastic modulus. However, these initial investigations revealed weak and minimal correlations, indicating that the properties did not exhibit meaningful covariance in their original dimensions.

To address this, PCA was employed to reduce the dimensionality of the dataset, which allowed for a better understanding of how these properties interact. The PCA results were remarkable, wherein the analysis captured 97.45% of the total variance, demonstrating the effectiveness of this statistical method. The PCA revealed that the first principal component (PC1) accounted for a significant proportion of the variance (84.97%), with most variables contributing moderately. This suggests that PC1 effectively represents a combination of properties that collectively influence the physical characteristics of silica aerogels.

One of the most notable findings was the strong positive correlation between PC1 and the weight percentage of silica (R^2^ = 94%). This correlation indicates that an increase in silica content is associated with a decrease in thermal conductivity, porosity, and BET surface area, while the density and elastic modulus increase. These findings underscore the importance of silica content in modulating the key physical properties of aerogels, making it a critical factor in their application and performance.

In contrast, the second principal component (PC2) exhibited a weaker correlation with the silica content (R^2^ = 3.87%), suggesting that it captures variations in thermal conductivity and other properties that are less dependent on silica percentage. However, when the low- or no-silica samples were excluded, the correlation between PC2 and silica content improved significantly (R^2^ = 81%), indicating that PC2 becomes more relevant at moderate to high silica levels.

Overall, the PCA findings emphasize the complex interplay between silica content and the physical properties of aerogels. This analysis highlights the potential of PCA, not only as a tool for dimensionality reduction but also as a means to uncover critical insights into the optimization of physical and chemical conditions for silica aerogel composites. These findings provide a valuable foundation for further research and development in the field, particularly in designing aerogels for specific industrial applications.

## 4. Materials and Methods

### 4.1. Principal Machine Learning Model for Exploring Cross-Correlation among Studied Features

To examine the cross-correlation among the various physico-chemical properties of silica aerogel, we employed a comprehensive machine learning (ML) model [45]. The dataset, comprising features such as ‘wt.% vs. silica’, ‘BET’, ‘pore volume’, ‘average pore diameter’, ‘density’, ‘thermal conductivity’, ‘porosity’, and ‘elastic modulus’, was initially loaded. Missing values were handled using mean imputation with the SimpleImputer program from scikit-learn. We conducted exploratory data analysis to understand the distribution and relationships between features, employing histogram plotting and scatter-plot matrices. These matrices visually depict pairwise correlations, providing initial insights into feature relationships. Pearson’s correlation coefficient was computed to quantify the linear relationships between features.

The formula for Pearson’s correlation coefficient (***r***) is:

(1) $$r=\frac{\sum \left(x_{i}-\bar{x}\right)\left(y_{i}-\bar{y}\right)}{\sqrt{\sum {\left(x_{i}-\bar{x}\right)}^{2}\sum {\left(y_{i}-\bar{y}\right)}^{2}}}$$

where $x_{i}$ and $y_{i}$ are the individual sample points. $\bar{x}$ and $\bar{y}$ are the means of the ***x*** and ***y*** features, respectively.

This ML approach allowed us to systematically investigate the cross-correlation among the features studied in this work, highlighting significant relationships and guiding further analysis.

### 4.2. Data Collection and Pre-Treatment

Data normalization is a critical step before performing PCA, due to its impact on the accuracy and interpretability of the results [46]. PCA is a technique used to reduce the dimensionality of datasets while preserving as much variance as possible [47]. However, PCA is sensitive to the scales of the variables in the dataset [48]. Without normalization, variables with larger scales can dominate the principal components, skewing the analysis and potentially leading to misleading conclusions [49]. Normalization ensures that each variable contributes equally to the analysis by rescaling the data to a common range, typically through methods such as standardization (subtracting the mean and dividing by the standard deviation; Equation (1)) [50]. This process allows PCA to identify the true underlying structure and patterns within the data being studied. Normalized data enhances the robustness of PCA by ensuring that the principal components represent a balanced combination of all variables, facilitating more accurate insights and reliable interpretations [51]. Therefore, data normalization is an essential preparatory step in PCA, ensuring that the results are meaningful and reflect the intrinsic relationships within the dataset.

Data were collected from the published study of Jadhav and Sarawade [14]. Table 1 presents the effect of the weight percentage of silica particles on the physical and operational features of aerogels where the precursor is tetraethyl orthosilicate (TEOS) and the reinforcing agent is glass fiber [6].

The data for each of the investigated variables has different weights. To eliminate any bias yielded by the difference in magnitude, a normalization technique similar to the one used by Younes et al. [27] has been adopted, as follows:

(2) $$Y_{st}=\frac{\left(Value-Mean\right)}{Standard\;Deviation}$$

where “*Y_st_*” represents the standardized dataset values.

### 4.3. Principal Component Analysis (PCA)

PCA serves as both a statistical and machine learning tool, offering versatile applications across various domains [52]. Statistically, PCA is used to reduce the dimensionality of datasets, which simplifies the complexity while preserving as much variance as possible. This reduction is achieved by transforming the original variables into a new set of uncorrelated variables called principal components (PCs), ordered according to the amount of variance they capture from the data [51]. PCs present an orthogonal position with each other, meaning that they exhibit independence. Hence, this makes PCA a suitable method to reveal the patterns between intercorrelated datasets [53]. This transformation facilitates easier visualization, interpretation, and understanding of data structures and relationships, which is particularly useful in fields like genetics [54], finance [55], and social sciences [56].

In the context of machine learning, PCA is often employed as a preprocessing step to enhance model performance [57]. By reducing the number of features, PCA helps mitigate issues like multicollinearity and overfitting, thereby improving the efficiency and accuracy of various algorithms, including regression models, clustering algorithms, and classification systems [58]. For instance, in image recognition and computer vision, PCA can reduce the dimensionality of pixel data, leading to faster and more accurate image analysis [59]. Additionally, PCA aids in noise reduction by focusing on the most significant components and discarding less relevant information [59]. This dual functionality as both a statistical and machine learning tool underscores PCA’s importance in data analysis, enabling more effective data-driven decisions and innovations [57,59].

After normalization, the PCA findings were generated using XLSTAT 2014 software, following a similar approach to that adopted by Younes et al. [28]. In this study, the missing data were omitted using a built-in feature that replaces a missing value with “Mode”, following the respective variables. The aim of the study is to apply PCA to the data found in a previous study by Jadhav and Sarawade [14], with a special emphasis on silica aerogels (Table 1). Applying PCA aims to uncover any hidden relationships between the physical/chemical properties and operational parameters [28,29,30]. If such relationships are found, this will enhance the interpretation and understanding of various factors that influence the applicability of a particular aerogel membrane. The insights gained from PCA can assist in multiple stages of the water treatment process, from manufacturing methods and experimental conditions to the removal efficiency of the chosen membrane [28,29,30]. Here, we have applied PCA to seven different factors, highlighting the effect of a particular weight percentage of added silica (6 wt.% silica aerogel samples) (Table 1). The procedure can be represented as follows:

(3) $$Fi=U_{j}^{T}M=\sum^{i=0}U_{ji}M_{i}$$

where *U* is the loading coefficient and *M* is the data vector of size *n*. The variance matrix *M*(*Var*(*M*)) is obtained by projecting *M* to *U*, which should be maximized as follows:

(4) $$Var\left(M\right)=\frac{1}{n}\left(UM\right){\left(UM\right)}^{T}=\frac{1}{n}{UMM}^{T}U$$

(5) $$MaxVar\left(M\right)=Max\left(\left(\frac{1}{n}\right)\;{UMM}^{T}U\right)$$

Since $\frac{1}{n}{MM}^{T}$ is the same as the covariance matrix of *M*(*cov*(*M*)), *Var*(*M*) can be expressed as follows:

(6) $$Var\;\left(M\right)=U^{T}cov\;\left(M\right)\;U$$

The Lagrangian function can be defined by performing the Lagrange multiplier method, as follows:

(7) $$L=U^{T}$$

(8) $$L=U^{T}cov\left(M\right)U-\delta (U^{T}U-1)$$

For (7), “*U^T^U* − 1” is considered to be equal to zero since the weighting vector is a unit vector. Hence, the maximum value of *Var*(*M*) can be calculated by equating the derivative of the Lagrangian function (*L*), with respect to *U*, as follows:

(9) $$\frac{dL}{dU}=0$$

(10) $$cov\left(M\right)U-\delta U=\left(cov\left(M\right)-\delta I\right)U=0$$

where *δ*: eigenvalue of *cov*(*M*); *U*: eigenvector of *cov*(*M*).

## Figures and Tables

**Table 1:** Effect of the weight percentage of silica particles on the physical and operational features of aerogels (the precursor is tetraethyl orthosilicate (TEOS), and the reinforcing agent is glass fiber) [14].

Wt.% of Silica Particles	Drying Technique	BET Specific Surface Area (m^2^/g)	Pore Volume (cm^3^/g)	Average Pore Diameter (nm)	Density (g/cm^3^)	Thermal Conductivity (W/mK)	Porosity (%)	Elastic Modulus (kPa)	References
0	CO_2_ SCD ^a^	1135.4	4.27	9.6	0.226	0.0283	91.3	1040	[6]
1	CO_2_ SCD ^a^	1077.4	3.94	8.3	0.231	0.0253	90.4	1280	[6]
3	CO_2_ SCD ^a^	985.86	3.77	8.1	0.236	0.0189	89.6	1500	[6]
3.5	APD ^b^	893.773	2.549	2.984	0.131	-	85.15	-	[22]
5	CO_2_ SCD ^a^	962.6	3.67	7.9	0.246	0.0179	89.3	1530	[6]
7	CO_2_ SCD ^a^	947.73	3.56	7.9	0.256	0.0194	88.7	1600	[6]
9	CO_2_ SCD ^a^	762.55	3.49	8.1	0.271	0.0219	87.2	1700	[6]
10	APD ^b^	593.051	2.193	3.848	0.152	-	83.76	-	[22]

^a^ SCD: supercritical drying. ^b^ APD: ambient pressure drying.

## Data Availability

The data presented and treated in this study are available from “Recent Advances Prospective of Reinforced Silica Aerogel Nanocomposites and Their Applications” [DOI: https://doi.org/10.1016/j.eurpolymj.2024.112766].
